# Analysis of Skin Pigmentation and Genetic Ancestry in Three Subpopulations from Pakistan: Punjabi, Pashtun, and Baloch

**DOI:** 10.3390/genes12050733

**Published:** 2021-05-13

**Authors:** Muhammad Adnan Shan, Olivia Strunge Meyer, Mie Refn, Niels Morling, Jeppe Dyrberg Andersen, Claus Børsting

**Affiliations:** 1 Section of Forensic Genetics, Department of Forensic Medicine, Faculty of Health and Medical Sciences, University of Copenhagen, 2100 Copenhagen, Denmark; olivia.meyer@sund.ku.dk (O.S.M.); mrefn@sund.ku.dk (M.R.); niels.morling@sund.ku.dk (N.M.); jeppe.dyrberg.andersen@sund.ku.dk (J.D.A.); claus.boersting@sund.ku.dk (C.B.); 2 Centre for Applied Molecular Biology (CAMB), University of the Punjab, Lahore 54590, Pakistan

**Keywords:** biogeographic ancestry, pigmentation, skin color, forensic DNA phenotyping, externally visible characteristics

## Abstract

Skin pigmentation is one of the most prominent and variable phenotypes in humans. We compared the alleles of 163 SNPs and indels from the Human Pigmentation (HuPi) AmpliSeq™ Custom panel, and biogeographic ancestry with the quantitative skin pigmentation levels on the upper arm, lower arm, and forehead of 299 Pakistani individuals from three subpopulations: Baloch, Pashtun, and Punjabi. The biogeographic ancestry of each individual was estimated using the Precision ID Ancestry Panel. All individuals were mainly of mixed South-Central Asian and European ancestry. However, the Baloch individuals also had an average proportion of Sub-Saharan African ancestry of approximately 10%, whereas it was <1% in the Punjabi and Pashtun individuals. The pairwise genetic distances between the Pashtun, Punjabi, and Baloch subpopulations based on the ancestry markers were statistically significantly different. Individuals from the Pashtun subpopulation had statistically significantly lower skin pigmentation than individuals from the Punjabi and Baloch subpopulations (*p* < 0.05). The proportions of European and Sub-Saharan African ancestry and five SNPs (rs1042602, rs10831496, rs1426654, rs16891982, and rs12913832) were statistically significantly associated with skin pigmentation at either the upper arm, lower arm or forehead in the Pakistani population after correction for multiple testing (*p* < 10^−3^). A model based on four of these SNPs (rs1426654, rs1042602, rs16891982, and rs12913832) explained 33% of the upper arm skin pigmentation. The four SNPs and the proportions of European and Sub-Saharan African ancestry explained 37% of the upper arm skin pigmentation. Our results indicate that the four likely causative SNPs, rs1426654, rs1042602, rs16891982, and rs12913832 located in *SLC24A5, TYR, SLC45A2,* and *HERC2,* respectively, are essential for skin color variation in the admixed Pakistani subpopulations.

## 1. Introduction

Human pigmentation is one of the most variable externally visible characteristics (EVCs). Prediction of EVCs may result in helpful leads early in a police investigation by providing a ‘genetic eye witness’ of a possible perpetrator and may allow the police investigators to focus their attention on specific groups of individuals and decrease the number of potential suspects [1,2,3]. The genetics of human pigmentation is a field of great interest in forensic genetics because it offers a prediction of three prominent EVCs: hair, eye, and skin color [4,5]. Skin pigmentation is strongly associated with biogeographic ancestry [6] and influenced by environmental factors (e.g., UV radiation) [7], which complicates the identification of causative variants and the reliability of the predictions. Prediction of the biogeographic ancestry may indicate skin pigmentation of the individual. However, skin color prediction based on the assumption that biogeographic ancestry and skin color are well correlated only offers an indirect prediction, and the assumption is not necessarily true in admixed populations [8,9]. Studies of admixed individuals from North and South American populations showed that the correlation between ancestry and skin pigmentation varied considerably and explained 20–65% of the skin pigmentation [3,6,10,11]. Nevertheless, admixed populations are ideal for studying the effect of genetic variants associated with skin pigmentation because they have a wide range of skin colors, and the ancestry proportions of each individual may be estimated using standard population genetic methods.

The Pakistani population is an admixed population with several subpopulations and ethnic groups. Pakistan is situated at the junction of the Middle East, Central Asia, and Southeast Asia. This area was the home of various ancient cultures, including the Bronze Age Indus Valley Civilization [12]. The region was ruled by dynasties and empires of different religions and cultures, including Muslims, Turco-Mongols, Hindus, Indo-Greeks, Sikhs, and Afghans [13]. Hence, the Pakistani population is well suited for studying biogeographic ancestry and skin pigmentation genetics.

We studied the associations among pigmentary variants, biogeographic ancestry, and quantitative skin pigmentation in three admixed subpopulations from Pakistan (*n* = 299). We evaluated the individuals’ ancestry with the Precision ID Ancestry Panel (Thermo Fisher Scientific, Waltham, MA, USA) [14,15,16] and investigated the association between skin pigmentation and genetic pigmentary variants in the HuPi AmpliSeq™ Custom panel [17]. We also analyzed the correlation between skin pigmentation and the 36 HIrisPlex-S SNPs [18,19] as well as nine SNPs found to be associated with skin pigmentation variation in an admixed Brazilian population [3]. Lastly, we analyzed the efficiency of the skin color prediction using the HIrisPlex-S prediction model [18,19].

## 2. Materials and Methods

### 2.1. Samples, DNA Extraction, and DNA Quantification

Buccal swabs were collected from 299 unrelated healthy Pakistani individuals and stored on FTA cards. The individuals belonged to three major Pakistani subpopulations: 107 Baloch from Baluchistan, 103 Pashtuns from Khyber Pakhtunkhwa, and 89 Punjabis from northern Punjab. Signed informed consent was obtained from all participants, and the samples were anonymized. The study was approved by the Review Board/Ethical Committee of the University of the Punjab, Pakistan (D/No. 019/DFEMS). Genomic DNA was extracted using the Qiagen BioRobot^®^ EZ1 Workstation and the EZ1 DNA investigator kit (Qiagen, Hilden, Germany). Purified DNA was quantified using the Qubit™ dsDNA HS Assay Kit and a Qubit^®^ 4.0 Fluorometer (Thermo Fisher Scientific, Waltham, MA, USA) according to the manufacturer’s recommendations.

### 2.2. Measurement of Quantitative Skin Pigmentation

The UV-Optimize Scientific 555 (Chromo Light APS, Vedbæk, Denmark) calibrated with a white standard (ISO 2469) was used to measure quantitative skin pigmentation. Quantitative skin pigmentation was determined as the pigment protection factor, PPF, which is a measure for skin pigmentation levels [20,21]. The skin pigmentation of each individual was measured in triplicates from skin areas on the lower inner forearm (lower arm), upper inner arm (upper arm), and forehead. The median PPF-value for each measuring site was used for further analysis. All skin measurements were taken from areas without hair, freckles, nevi, or tattoos.

### 2.3. Typing with the Precision ID Ancestry Panel and the Human Pigmentation (HuPi) AmpliSeq™ Custom Panel

DNA was amplified with the Precision ID Ancestry Panel (Thermo Fisher Scientific, Waltham, MA, USA) and the Human Pigmentation (HuPi) AmpliSeq™ Custom panel [17] in two separate reactions. The Precision ID Ancestry Panel targets 165 ancestry-informative markers (AIMs). The HuPi AmpliSeq™ Custom panel targets 183 SNPs and indels, which were previously found to be associated with human pigmentary variation [17]. Sequencing libraries were prepared with the Ion AmpliSeq™ Library Kit 2.0 (Thermo Fisher Scientific, Waltham, MA, USA) using half volume of all reagents but otherwise following the manufacturer’s instructions. Amplification of targets was carried out using approximately 1 ng DNA (1–3 µL) and 24 and 26 cycles for the Precision ID Ancestry Panel and the HuPi AmpliSeq™ Custom panel, respectively. Sequencing libraries were purified using the Biomek 3000 pipetting robot [22]. Purified libraries were quantified using the Qubit™ 4.0 Fluorometer with the Qubit™ dsDNA HS Assay kit (Thermo Fisher Scientific, Waltham, MA, USA) and pooled in equimolar concentrations to a final volume of 28 pM for the Precision ID Ancestry panel and 35–60 pM for the HuPi AmpliSeq™ Custom Panel. Template preparation was performed with the Ion Chef™ instrument (Thermo Fisher Scientific, Waltham, MA, USA) using the Ion S5™ Precision ID Chef Kit (Thermo Fisher Scientific, Waltham, MA, USA) following the manufacturer’s recommendations (Thermo Fisher Scientific, Waltham, MA, USA). Sequencing was carried out with Ion 530™ Chips and Ion S5™ Precision ID Sequencing Reagents on the Ion S5™ (Thermo Fisher Scientific, Waltham, MA, USA) with up to 80 samples per chip.

### 2.4. Analysis of Sequencing Data

The data were initially analyzed with the Torrent Suite Software v.5.10.1 (Thermo Fisher Scientific, Waltham, MA, USA). For the Precision ID Ancestry panel, data were analyzed with the HID_SNP_Genotyper_v5_2_2 plugin using GRCh37 (hg19) as the reference genome and default parameters (Thermo Fisher Scientific, Waltham, MA, USA). The data obtained with the HuPi AmpliSeq™ Custom Panel were analyzed with the VariantCaller v5.10.1.20 plugin and the GRCh38.p2 (hg38) reference genome. The “Down-sample to coverage” was changed from 400 to 10,000 reads. Otherwise, the analyses were performed with the default parameters (Thermo Fisher Scientific, Waltham, MA, USA). The resulting Excel-files were processed using R v.4.0.2 (R core team, https://www.R-project.org/, accessed on 1 November 2020) and an in-house developed script. The data quality control comprised evaluation of the locus balance, heterozygote balance (Hb), and noise levels for each target. Hb was calculated as the number of reads for one nucleotide divided by the number of reads for the other nucleotide in the order A, C, G, and T. Genotypes were accepted when a locus had ≥45 reads and the Hb was between 0.3 and 3. SNPs with 30–44 reads were inspected and accepted if the Hb was between 0.7 and 1.5 or if the noise did not exceed one read for homozygous genotype calls. Genotypes that did not meet the criteria were annotated as NN. For both panels, loci with NNs in more than 15% of the samples were excluded. Moreover, samples with more than 15% missing data (NN) were excluded from further analysis. Hardy–Weinberg Equilibrium (HWE) and pairwise linkage disequilibrium (LD) were calculated with Haploview v. 4.2 [23], and pairwise *F_ST_*-values were calculated using Arlequin v3.5.2.2 software [24].

### 2.5. Classification of Biogeographic Ancestry

The alleles of the AIM-SNPs in each sample were investigated using STRUCTURE v.2.3.4.21 [25,26]. The analyses were carried out using 100,000 steps of burn-in followed by 100,000 repetitions for the MCMC. The ‘admixture’ and the ‘correlated allele frequencies’ models were used [25,26]. After testing the number of clusters (*K*) from K = 3 to K = 6, K was set to 4, corresponding to the Sub-Saharan African, South-Central Asian, European, and East Asian populations. The results were visualized using CLUMPP v.1.1.222 [27] and Distruct v.1.1.23 [28]. Principal component analysis (PCA) was carried out using an in-house written Python script. Reference population data (Supplementary Table S1) were collected as previously described [14,29]. Four SNPs, rs1800414, rs12913832, rs1426654, and rs16891982, were included in both the Precision ID Ancestry Panel and the HuPi AmpliSeq™ Custom panel. One SNP, rs10954737, lacked genotype data in all reference populations. Hence, these five SNPs were not considered in the PCA and STRUCTURE analyses and were not used to classify ancestry.

### 2.6. Correlations between Biogeographic Ancestry, Pigmentary Variants, and Skin Pigmentation

We excluded monomorphic genetic variants (i.e., variants with only one observed genotype) and variants in LD (r^2^ > 0.8) with other variants in the HuPi AmpliSeq™ Custom Panel. Hence, the dataset was limited to 102 genetic variants and four metapopulations (Sub-Saharan Africa, South-Central Asia, Europe, and East Asia). Differences in skin pigmentation levels of the lower arm, upper arm, and forehead, as well as differences in skin pigmentation of the three subpopulations, were investigated using Student’s *t*-test for paired data or the Welch two-sample *t*-test for unpaired data. The correlations among skin pigmentation levels, genetic variants in the HuPi AmpliSeq™ Custom Panel, and the estimation of the ancestry proportions were carried out using multiple linear regression (MLR). The correlation was evaluated using the adjusted R^2^. To select a subset of variants, backwards model selection was performed using MLR with Akaike Information Criterion (AIC) using the *stepAIC* function in the MASS R-package [30].

We also evaluated the correlation between skin pigmentation and the genotypes of the 36 HIrisPlex-S SNPs [18,19], and between skin pigmentation and nine SNPs (rs1426654, rs1448484, rs16891982, rs4424881, rs10831496, rs6119471, rs12913832, rs10424065, and rs1408799) previously found to explain up to 65% of the skin pigmentation variation in a Brazilian population of primarily European ancestry [3].

### 2.7. Prediction of Skin Colour Using the HIrisPlex-S

Prediction of skin color using the HIrisPlex-S model was carried out with the online web-tool (https://hirisplex.erasmusmc.nl/, accessed on 1 Novmber 2020). The HIrisPlex-S model predicts skin color in five categories: Very pale, Pale, Intermediate, Dark, and Dark to Black [18,19]. The skin color category with the highest predictive value was used as the skin pigmentation prediction. The HIrisPlex-S skin color prediction and the quantitative skin pigmentation measurements were compared.

### 2.8. Statistical Methods

The statistical methods used are described in each section. When multiple comparisons were performed, the statistical significance was corrected with the Bonferroni method.

## 3. Results

### 3.1. Skin Pigmentation Measurements

Quantitative skin pigmentation measurements were performed for the 299 Pakistani individuals, Baloch (*n* = 107), Pashtun (*n* = 103), and Punjabi (*n* = 89). The lowest pigmentation levels were observed on the upper arm (mean PPF of 11.08), followed by the lower arm (mean PPF of 12.30) and the forehead (mean PPF of 12.43). We observed statistically significant differences between the pigmentation levels on the upper arm and lower arm (*p* < 0.05) and the upper arm and forehead (*p* < 0.05) among all subpopulations. Statistically significant difference between pigmentation levels of the lower arm and forehead was only observed in the Baloch subpopulation (*p* < 0.05).

**Figure 1:**
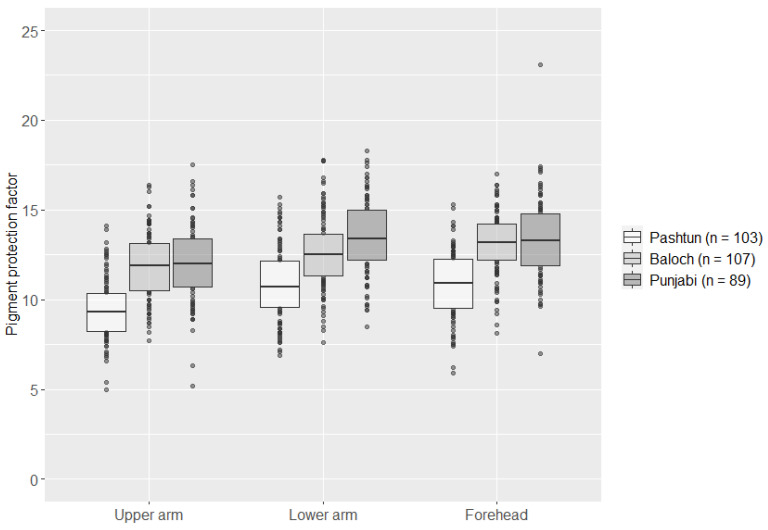
Boxplots of skin pigmentation measurements (pigment protection factor) of the upper arm, lower arm, and forehead in three Pakistani subpopulations. The pigment protection factor was measured in triplicate at each measured skin area.

Individuals from the Pashtun subpopulation showed statistically significant (*p* < 0.05) lower pigmentation levels than individuals in the Baloch and Punjabi subpopulations on all measured areas (Figure 1).

### 3.2. The Precision ID Ancestry Panel

The allele frequencies for the 165 loci in the Punjabi, Pashtun, and Baloch populations are presented in Supplementary Table S2. The median number of reads per target was 493 (range: 45–12,105), the median Hb was 1.0 (range: 0.3–3.0) for heterozygous SNPs, and the median level of noise was 0.0% (range: 0–14.3%). One locus, rs2070586, deviated from HWE (*p* < 10^−3^) in the Baloch population. The loci rs3811801 and rs671 were monomorphic in all three populations, rs1871534 and rs2814778 were monomorphic in Pashtuns and Punjabis, and rs1800414 was monomorphic in the Baloch. No pairwise LD (pairwise r^2^ > 0.8) was detected in any of the three populations. The observed values of pairwise *F_ST_* genetic distances were statistically significantly different among all subpopulations (*p <* 10^−5^) (Supplementary Table S3).

### 3.3. Proportion of Ancestry Components

**Figure 2:**
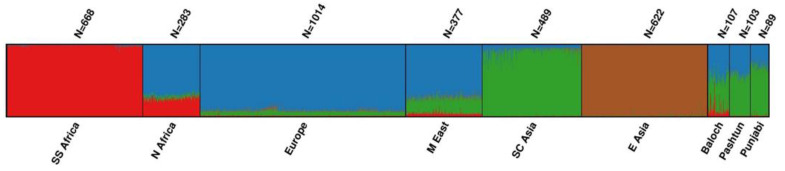
STRUCTURE analysis with K = 4 using 160 ancestry informative markers (AIMs) and six meta-populations. Each cluster (K) is represented by a color. Population abbreviations used: SS Africa: Sub-Saharan Africa; N Africa: North Africa; M East: Middle East; SC Asia: South-Central Asia; E Asia: East Asia.

The biogeographic ancestry of each individual was investigated with STRUCTURE and PCA using reference data from 34 populations grouped into six metapopulations: Sub-Saharan African, North African, European, Middle Eastern, South-Central Asian, and East Asian (Supplementary Table S1). The results of the STRUCTURE analysis with K = 4 are shown in Figure 2. The Baloch, Punjabi, and Pashtun subpopulations were admixed populations with major contributions from South-Central Asian and European populations. The Baloch differed from the other two subpopulations due to approximately 10% Sub-Saharan African genetic contribution, whereas the Sub-Saharan African contribution was <1% in the Punjabis and Pashtuns. The Baloch had the lowest proportion of South-Central Asian ancestry (44.7%), followed by the Pashtuns (56.6%) and the Punjabis (69.3%).

The first two principal components (PC1 and PC2) in the PCA analysis separated the Sub-Saharan African, European, and East Asian populations from each other (Supplementary Figure S1). The North Africans clustered closest to the European and Middle Eastern populations, while the South-Central Asian populations clustered between the East Asian and European populations. The positions of the three Pakistani subpopulations, Punjabis, Pashtuns, and the Baloch, overlapped with each other and the South-Central Asian and Middle Eastern populations. The Baloch individuals clustered closer to the African populations than the Pashtuns and Punjabis. In contrast, the Punjabis clustered closer to the South-Central Asian populations than the Pashtuns and Baloch, supporting the STRUCTURE analysis conclusions.

### 3.4. The HuPi AmpliSeq™ Custom Panel

A total of 163 variants were successfully typed with the HuPi AmpliSeq™ Custom Panel (Supplementary Table S4). The median number of reads was 597 (range: 60–1532) per target. The median Hb for heterozygous SNP calls was 1.0 (range: 0.35–2.86). The median level of noise was 0.0% (range: 0–12.7%). The allele frequencies of rs4778241 in *OCA2* deviated statistically significantly from HWE (*p* < 10^−3^). Twelve variants were monomorphic (Supplementary Table S4), and these data were removed from the subsequent analysis. For the correlation measurements between skin pigmentation and genetic variants, only independent genetic variants were considered (pairwise r^2^ < 0.8), reducing the number of genetic variants to 102 (Supplementary Table S4).

### 3.5. Correlation between Skin Pigmentation and Biogeographic Ancestry

The proportions of Sub-Saharan African, European, South-Central Asian, and East Asian ancestry were compared with the skin pigmentation at each measured site using MLR. We found statistically significant (*p* < 10^−3^) adjusted R^2^-values for the upper arm: 0.10, lower arm: 0.13, and forehead: 0.12 when comparing the full ancestry profiles with the skin pigmentation (Supplementary Table S5). We found statistically significant (*p* < 0.05) negative correlations between the proportion of European ancestry and skin pigmentation at all three measured sites (adjusted R^2^: 0.04–0.09) and statistically significant positive correlation (*p* < 0.05) between Sub-Saharan African ancestry and skin pigmentation at the upper arm and forehead (adjusted R^2^ = 0.06 and 0.07, respectively). In contrast, the proportions of East Asian and South-Central Asian ancestry showed no statistically significant correlation with skin pigmentation (Supplementary Table S5). We analyzed the correlation between the proportions of only European and Sub-Saharan African ancestry with skin pigmentation at all three measuring sites using MLR. The resulting adjusted R^2^-values were 0.10 (*p* < 10^−3^), 0.13 (*p* < 10^−6^), and 0.12 (*p* < 10^−6^) for the upper arm, lower arm, and forehead pigmentation, respectively (Supplementary Table S5).

### 3.6. Correlations among Skin Pigmentation, Pigmentary Variants, and Biogeographic Ancestry

Each of the independent variants typed with the HuPi AmpliSeq™ Custom Panel was compared with the skin pigmentation at the upper arm, lower arm, and forehead using linear regression. We observed statistically significant (*p* < 0.05) correlations between skin pigmentation on either the upper arm, lower arm, or forehead and the SNPs rs1042602 in *TYR* and rs10831496 in *GRM5*, rs1426654 in *SLC24A5*, rs16891982 in *SLC45A2*, and rs12913832 in *HERC2* (Table 1). Only rs1426654 and rs1042602 were statistically significantly correlated with skin pigmentation at all three sites (*p* < 10^−3^) (Table 1).

**Figure 3:**
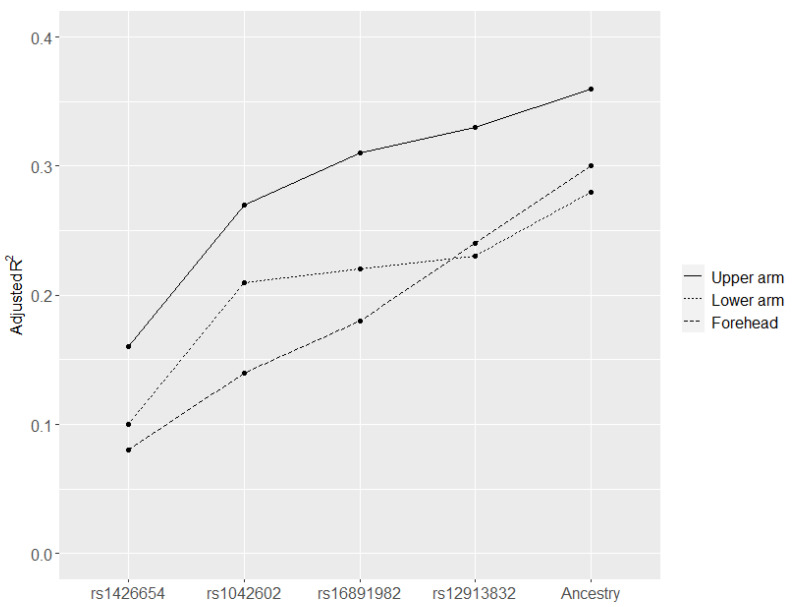
Cumulative correlations between (1) four SNPs and ancestry and (2) upper arm, lower arm, and forehead pigmentation. The SNPs were ranked based on their correlations with skin color (adjusted R^2^). Ancestry is the proportion of Sub-Saharan African and European ancestry.

The five SNPs were compared with skin pigmentation using MLR, and the adjusted R^2^ ranged from 0.24 to 0.33 (*p* < 10^−6^) (Table 2). Subsequently, model selection was performed using stepwise AIC, giving the best model with rs1426654, rs1042602, rs16891982, and rs12913832 for all three sites (upper arm, lower arm, and forehead). When the proportions of European and Sub-Saharan African ancestry were included, the adjusted R^2^-values increased slightly (Table 2, Figure 3). The cumulative correlations between (1) the four SNPs and ancestry and (2) the skin pigmentation are shown in Figure 3. The four SNPs, ancestry, and the upper arm pigmentation showed the highest adjusted R^2^-values (Table 2 and Figure 3).

### 3.7. Correlation of Skin Pigmentation with SNPs from Existing Skin Colour Models

The skin pigmentation in the Pakistani individuals was compared with nine SNPs previously found to be associated with skin pigmentation in a Brazilian population of primarily European ancestry [3]. The nine SNPs included rs10831496, rs12913832, rs1426654, and rs16891982, which were associated with skin pigmentation in the Pakistani population (Table 1), as well as rs1448484 in *OCA2*, rs4424881 in *APBA2*, rs6119471 in *ASIP*, rs1408799 in *TYRP1*, and rs10424065 in *MFSD12,* which were not associated with skin pigmentation in our study. In the Pakistani population, these nine SNPs explained up to 26% of the skin pigmentation (upper arm). The adjusted R^2-^values were 0.26 (*p <* 0.05), 0.15 (*p <* 0.05), and 0.19 (*p <* 0.05) for the upper arm, lower arm, and forehead pigmentation, respectively.

We also correlated the HIrisPlex-S SNPs with the skin pigmentation on the upper arm, lower arm, and forehead using MLR. Four SNPs in the *MC1R*-gene, rs3212355, rs1805006, rs11547464, and rs1110400 were monomorphic and excluded from the analysis. Thus, the correlation was based on 32 of the 36 skin color predictive SNPs in the HIrisPlex-S. The adjusted R^2^-values were 0.36 (*p <* 10^−6^), 0.29 (*p* < 10^−6^), and 0.27 (*p* < 10^−6^), for pigmentation on the upper arm, lower arm, and forehead, respectively.

**Figure 4:**
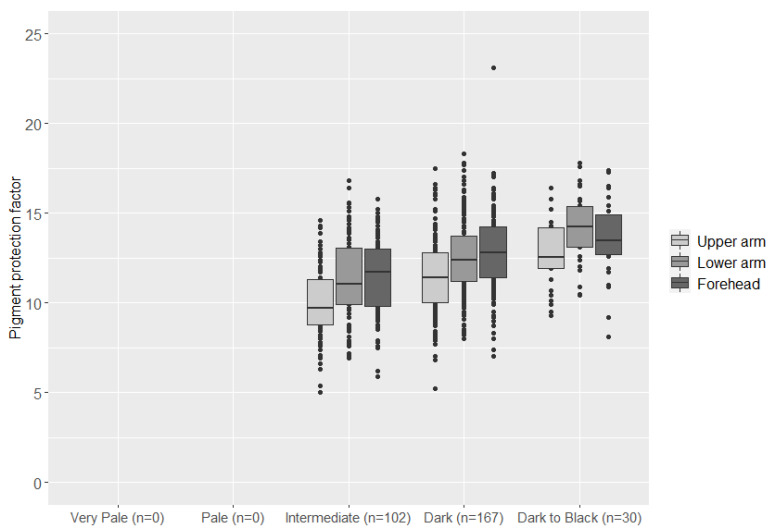
Boxplots showing the skin color category predicted with the HIrisPlex-S prediction model and the pigmentation protection factor on the upper arm, lower arm, and forehead.

Lastly, we evaluated the performance of the HIrisPlex-S model that is a forensically validated skin color prediction model [18,19]. The predicted skin colors of the 299 Pakistani individuals were: Intermediate: 102, Dark: 167, and Black: 30. No individual was predicted to have very pale or pale skin colors. Comparisons between the predicted skin color categories and the quantitative skin pigmentation measurements are shown in Figure 4.

## 4. Discussion

The pairwise genetic distances calculated among the three subpopulations were statistically significantly different (Supplementary Table S3). The Baloch were more distant from Punjabis and Pashtuns than Punjabis were from Pashtuns. The results agreed with our previous results obtained with short tandem repeats that depicted genetic differences between the Baloch and other subpopulations from Pakistan [31]. The STRUCTURE analysis (Figure 2) showed that all three subpopulations from Pakistan were admixed, mainly with South-Central Asian and European genetic contributions. However, the Baloch population also had a genetic contribution from Sub-Saharan African populations, which may be remnants of African individuals settling in the Indian subcontinent [32,33].

The proportion of European and Sub-Saharan African ancestry affected the skin pigmentation, whereas the proportion of South-Central Asian ancestry did not (Supplementary Table S5). However, the proportions of European and Sub-Saharan African ancestry only explained approximately 10% of the variation (Supplementary Table S5) and only influenced the adjusted R^2^-values of the MLR model marginally (Table 2). This showed that ancestry was a relatively poor predictor of skin pigmentation in the highly admixed Pakistani populations. It also indicated that any association between a locus and skin pigmentation levels would most likely come from causative SNPs or indels, and not be a consequence of population genetic differences.

Of the 163 variants that were previously shown to be associated with pigmentary traits, five SNPs were statistically significantly correlated with the skin pigmentation levels of the investigated individuals from the Pakistani subpopulations (Table 1). These five SNPs are located in five genes, *TYR, GRM5, SLC24A5*, *SLC45A2,* and *HERC2.* Four of the five SNPs, rs1042602 in *TYR*, rs1426654 in *SLC24A5*, rs16891982 in *SLC45A2*, and rs12913832 in *HERC2*, explained 33% of the skin pigmentation variation on the upper arm area and 23–24% of the skin pigmentation variation on the lower arm and forehead that are less protected from UV irradiation (Table 2). Three of the SNPs, rs1426654 (ranked 1), rs1042602 (ranked 2), and rs16891982 (ranked 3), were previously found to be associated with skin pigmentation in individuals of South Asian descent [7,34,35]. In agreement with our results, the *SLC24A5* SNP rs1426654 had the most pronounced effect on skin pigmentation levels [7,34,36]. The *HERC2* SNP rs12913832 (ranked 4) has not previously reported to be associated with skin pigmentation in South Asian populations. However, it was reported to be associated with skin color in other populations [11,19]. The fifth SNP, *GRM5* rs10831496 (ranked 5), did not increase the cumulative adjusted R^2^-value of the MLR model at any of the measured pigmentation areas. This SNP is highly polymorphic and located in intron 3 of the *GRM5* gene, 340 kbp upstream of the *TYR* gene on chromosome 11. *GRM5* rs10831496 was previously reported to be associated with tanning response after exposure to sunlight [37]. In our study, it was associated with skin pigmentation on the lower arm.

The three highest ranking SNPs, rs1426654, rs1042602, and rs16891982, are non-synonymous variants: Thr111Ala in *SLC24A5*, Ser192Tyr in *TYR*, and Phe374Leu in *SLC45A2*, respectively, and they all influence melanin production. In human primary melanocytes that were homozygous for the *SLC24A5* 111Ala variant, the TYR activity, melanin content, and the number of mature melanosomes were decreased [38]. The 192Tyr *TYR* variant reduced the TYR activity by 40% [39] and the number of primary melanocytes, and human skin tissue samples that were homozygous for the 192Tyr variant had reduced TYR activity and expression [40]. The *SLC45A2* 374Leu variant was found in increased frequency in non-European populations, and 374Leu homozygous melanocytes had higher TYR activity and melanin content than melanocytes that were homozygous for the 374Phe variant [38]. The fourth-ranking SNP, *HERC2* rs12913832, is the most important variant for eye color determination [41,42,43]. It is positioned in a key enhancer element of the *OCA2* pigmentary gene [44]. Human primary melanocytes that were homozygous for the derived rs12913832 G allele had reduced TYR activity, melanin content, and numbers of mature melanosomes [38]. When we analyzed five additional SNPs that were previously found to be associated with pigmentation in an admixed Brazilian population with primarily European and Sub-Saharan African genetic contributions [3], the correlation with skin pigmentation of the upper arm was reduced from 33% to 26%. This indicates that the biogeographic background of an individual is important for the selection of markers that eventually will give the best prediction of skin color, which is well in line with the knowledge that some genetic markers are associated with skin pigmentation in certain populations and not in others [45]. It also indicates that the estimation of the biogeographic background should be the first step in the estimation of skin pigmentation levels from DNA samples of unknown individuals. The second step should be to investigate causative variants found to be associated with skin pigmentation in the most likely biogeographic background of the collected trace sample and these variants should form the basis for the estimation of skin pigmentation.

In forensics genetic casework, ancestry inference can provide helpful information about an unidentified perpetrator who left a biological trace sample at a crime scene. Several well-established AIM panels that successfully differentiate the major human populations, including Europeans, East Asians, South Central Asians, Native Americans, Sub-Saharan Africans, and Oceanians, have been developed [14,46,47,48,49]. Ancestry information may also provide indications on EVCs that are typical for a given population. However, caution must be taken when using ancestry to infer the phenotypes of an individual. An individual of mixed ancestry may have AIM alleles inherited from one parental population, but phenotypic characteristics, e.g., skin color, of the other parental population [3,9]. Thus, skin color and other EVCs should be estimated using causative genetic variants. We identified four likely causative variants that influence skin pigmentation in Pakistanis and are included in the leading skin color prediction model, HIrisPlex-S [18,19]. However, the variants only explained approximately one-third of the skin pigmentation variation and the remaining SNPs of the HIrisPlex-S only slightly increased the adjusted R^2^-values underlining the need for additional genetic studies of human skin pigmentation.

## Figures and Tables

**Table 1:** Five SNPs with statistically significant association with skin pigmentation on the upper arm, lower arm, or forehead in 299 Pakistani individuals from three subpopulations (Baloch, Pashtun, and Punjabi).

		Upper Arm		Lower Arm		Forehead	
Gene	SNP	Adjusted R^2^	Sign.^1^	Adjusted R^2^	Sign. ^1^	Adjusted R^2^	Sign. ^1^
*TYR*	rs1042602	0.13	***	0.11	**	0.071	**
*GRM5*	rs10831496	0.029	–	0.047	*	0.014	–
*HERC2*	rs12913832	0.025	–	0.016	–	0.064	*
*SLC24A5*	rs1426654	0.16	***	0.10	**	0.082	**
*SLC45A2*	rs16891982	0.075	**	0.028	–	0.058	**

^1^ Statistical significance (Sign.) after Bonferroni correction: * *p* < 0.05, ** *p* < 10^−3^, *** *p* < 10^−6^.

**Table 2:** Multiple loci correlations with skin pigmentation on the upper arm, lower arm, or forehead in 299 Pakistani individuals from three subpopulations (Baloch, Pashtun, and Punjabi).

	Upper Arm		Lower Arm		Forehead	
	Adjusted R^2^	Sign. ^1^	Adjusted R^2^	Sign. ^1^	Adjusted R^2^	Sign. ^1^
Five SNPs ^2^	0.33	***	0.24	***	0.24	***
Best model ^3^	0.33	***	0.23	***	0.24	***
Best model + ancestry ^4^	0.37	***	0.28	***	0.30	***

^1^ Statistical significance (Sign.): *** *p* < 10^−6^. ^2^ rs1042602, rs10831496, rs12913832, rs1426654, and rs16891982. ^3^ rs1042602, rs12913832, rs1426654, and rs16891982. ^4^ Ancestry is the estimated proportion of European and Sub-Saharan African ancestry.

## Data Availability

The data generated in the present study are included within the manuscript and its supplementary file.

## Supplementary Materials

The following are available online at https://www.mdpi.com/article/10.3390/genes12050733/s1, Figure S1: PCA plot of the studied populations and selected reference populations, Table S1: Reference data used in the population comparison analysis, Table S2: Allele frequencies of 165 variants typed with the Precision ID Ancestry panel in 299 Pakistani individuals from three subpopulations (Baloch, Pashtun, and Punjabi), Table S3: Pairwise FST-values among the three Pakistani subpopulations Baloch, Pashtun, and Punjabi based on the allele frequencies of 165 AIMs typed with the Precision ID Ancestry panel, Table S4: Allele frequencies of 163 variants typed with the Human Pigmentation (HuPi) AmpliSeq™ Custom panel in 299 Pakistani individuals from three subpopulations (Baloch, Pashtun, and Punjabi), Table S5: Estimated ancestry proportions associated with skin pigmentation on the upper arm, lower arm, or forehead in 299 Pakistani individuals from three subpopulations (Baloch, Pashtun, and Punjabi).
