# Supplementary material for: Analysis of Skin Pigmentation and Genetic Ancestry in Three Subpopulations from Pakistan: Punjabi, Pashtun, and Baloch
Source: Genes (Basel). 2021 May 13;12(5):733. doi: 10.3390/genes12050733 (PMC8152963; doi:10.3390/genes12050733)
Supplement: Supplementary file 1 [file genes-12-00733-s001.zip › genes-1198547-SI/genes-1198547-SI.pdf]

SUPPLEMENTARY MATERIAL

Analysis of skin pigmentation and genetic ancestry in three subpopulations from Pakistan: Punjabi, Pashtun, and Baloch

Muhammad Adnan Shan[#] [a,b], Olivia Strunge Meyer[#] [a], Mie Refn[a], Niels Morling[a], Jeppe Dyrberg Andersen* [a], Claus Børsting[a]

[a] Section of Forensic Genetics, Department of Forensic Medicine, Faculty of Health and Medical Sciences, University of Copenhagen, 2100 Copenhagen, Denmark
[b] Centre for Applied Molecular Biology (CAMB), University of the Punjab, 54590 Lahore, Pakistan

[#]These authors contributed equally.

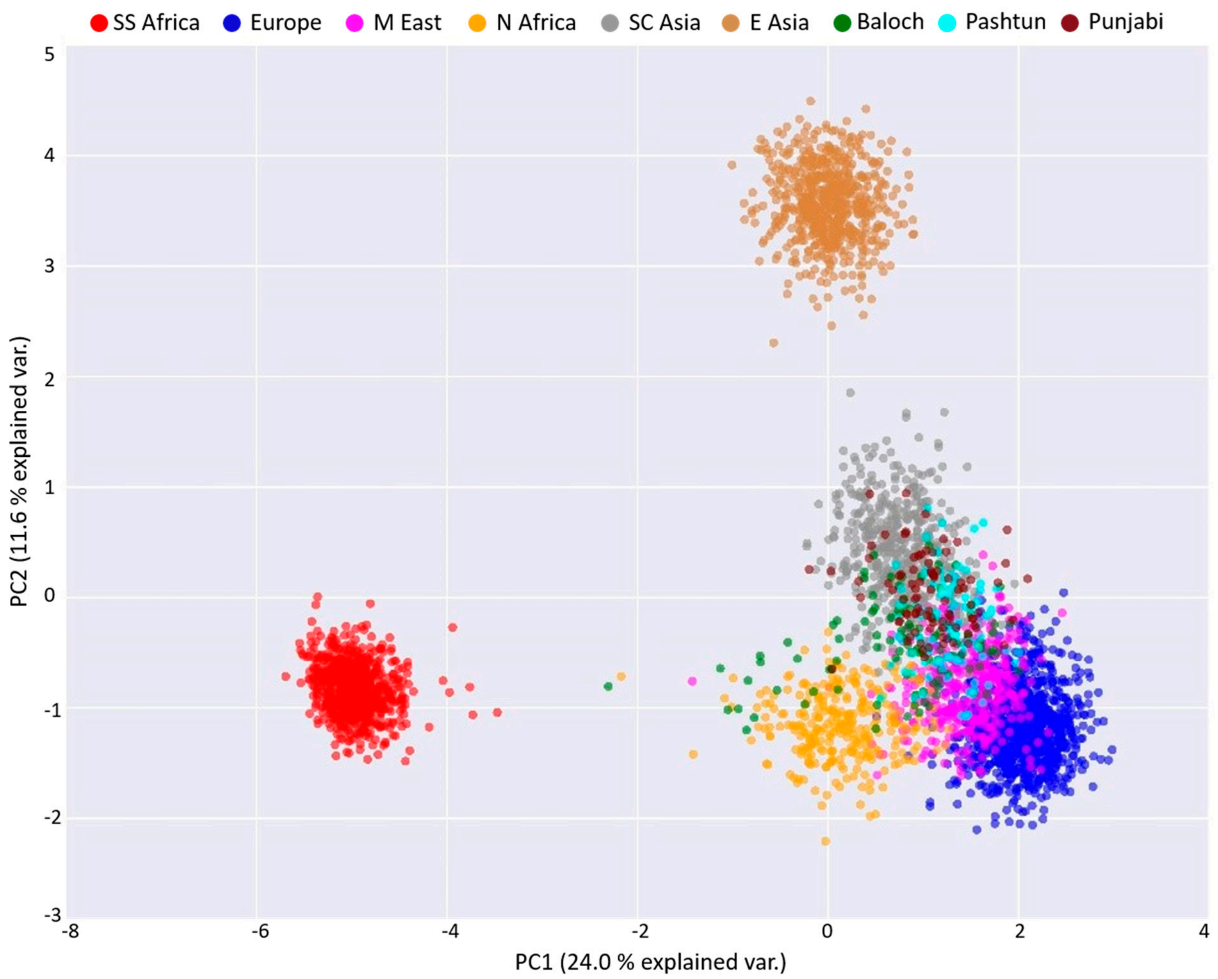

Figure S1: PCA plot of the studied populations and selected reference populations. Each coloured symbol represents an individual according to the population membership. Abbreviations used: SS Africa: Sub-Saharan Africa, M East: Middle East, N Africa: North Africa, SC Asia: South-Central Asia, E Asia: East Asia.

Table S1: Reference data used in the population comparison analysis.

Meta-population	Abbreviation	Description	N
**Sub-Saharan Africa**	GWD	Gambians, Western Division,The Gambia[†]	113
	MSL	Mende, Sierra Leone[†]	85
	ESN	Esan, Nigeria[†]	99
	YOR	Yoruba, Benin City, Nigeria[†]	77
	YRI	Yoruba, Ibadan, Nigeria[†]	108
	NIG	Nigeria (samples from 2 locations)[a,b†]	87
	LWK	Luhya, Webuye, Kenya[†]	99
**North Africa**	TUN	Tunisia (samples from 7 locations)[a,c†]	208
	MOR	Morocco RGA*	75
**Europe**	IBS	Iberians, Spain[†]	107
	TSI	Toscani, Italy[†]	107
	HGR	Hungarians[†]	89
	EAM	European Americans[†]	89
	GBR	British from England and Scotland[†]	91
	CEU	Utah Residents (CEPH), N&W Europe ancestry[†]	99
	IRI	Irish[†]	113
	FN1	Finns, Finland[†]	99
	DK	Denmark RGA*	140
	RUS	Russia (samples from 2 locations)[a,d†]	80
**Middle East**	DRU	Druze, Israel[†]	102
	IRQ	Iraq RGA*	94
	IRN	Iran RGA*	93
	TUR	Turkey RGA*	88
**South-Central Asia**	PJL	Punjabi, Lahore, Pakistan[†]	96
	GIH	Gujarati (India), Houston, Texas[†]	103
	ITU	Telugu (India), collected in United Kingdom[†]	102
	STU	Tamil (Sri Lanka), collected in United Kingdom[†]	102
	BEB	Bengali, Bangladesh[†]	86
**East Asia**	JPT	Japanese, Tokyo, Japan[†]	104
	CHB	Han Chinese in Bejing, China[†]	103
	HCS	Southern Han Chinese[†]	105
	CDX	Chinese Dai in Xishuangbanna, China[†]	93
	KHV	Kinh (Vietnamese), Ho Chi Minh City, Vietnam[†]	99
	LAO	Laotians[†]	118
**Studied**	BAL	Baloch, Pakistan	107
	PAS	Pashtuns, Pakistan	103
	PUN	Punjabis, Pakistan	89

[†]Reference data were collected in the Kidd Lab and assembled by the Kidd Lab from other publicly available. The Kidd lab had no further involvement in data analysis.

[*]RGA: Section of Forensic Genetics, Department of Forensic Medicine, Faculty of Health and Medical Sciences, University of Copenhagen.

[a]There were no statistically significant differences between the allele and genotype frequencies of the population samples.

[b]Ibo and Hausa.

[c]Nebeur, Kesra, Kairoun, Sousse, Mehdia, Kerkennah and Smar.

[d]Archangelsk and Vologda.

Table S2: Allele frequencies of 165 variants typed with the Precision ID Ancestry panel in 299 Pakistani individuals from three subpopulations (Baloch, Pashtun, and Punjabi).

	Minor allele frequency			
**Target ID**	**Baloch (n = 107)**	**Pashtun (n = 103)**	**Punjabi (n = 89)**	**Alleles (major : minor)**
rs10007810	0.314	0.347	0.330	G:A
rs10108270	0.363	0.379	0.444	C:A
rs10236187	0.201	0.063	0.107	A:C
rs1040045	0.201	0.097	0.129	A:G
rs1040404	0.285	0.248	0.202	G:A
rs10496971	0.175	0.275	0.247	T:G
rs10497191	0.206	0.117	0.118	C:T
rs10511828	0.080	0.094	0.045	T:C
rs10512572	0.195	0.098	0.157	G:A
rs10513300	0.098	0.078	0.118	T:C
rs1079597	0.159	0.184	0.208	C:T
rs10839880	0.369	0.353	0.360	T:C
rs10954737	0.079	0.102	0.073	T:C
rs11227699	0.075	0.034	0.051	G:A
rs11652805	0.311	0.291	0.213	T:C
rs12130799	0.093	0.058	0.034	A:G
rs1229984	0.065	0.098	0.101	C:T
rs12439433	0.024	0.027	0.006	A:G
rs12498138	0.096	0.041	0.047	G:A
rs12544346	0.266	0.393	0.393	G:A
rs12629908	0.126	0.142	0.124	G:A
rs12657828	0.182	0.157	0.124	A:G
rs12913832	0.065	0.165	0.067	A:G
rs1296819	0.281	0.180	0.182	C:A
rs1325502	0.238	0.238	0.202	G:A
rs13400937	0.403	0.489	0.448	T:G
rs1369093	0.171	0.102	0.138	T:C
rs1407434	0.129	0.135	0.182	G:A
rs1426654	0.120	0.068	0.188	A:G
rs1462906	0.131	0.044	0.090	C:T
rs1471939	0.364	0.447	0.404	T:C
rs1500127	0.089	0.097	0.079	C:T
rs1503767	0.229	0.177	0.230	T:G
rs1513056	0.262	0.304	0.348	G:A
rs1513181	0.299	0.330	0.337	G:A
rs1569175	0.099	0.076	0.101	C:T
rs1572018	0.283	0.237	0.343	C:T
rs16891982	0.136	0.262	0.176	C:G
rs174570	0.118	0.078	0.084	C:T
rs1760921	0.150	0.189	0.107	T:C
rs17642714	0.159	0.180	0.163	A:T

Table S2 (continued)

	Minor allele frequency			
**Target ID**	**Baloch (n = 107)**	**Pashtun (n = 103)**	**Punjabi (n = 89)**	**Alleles (major : minor)**
rs1800414	0.000	0.010	0.011	T:C
rs1834619	0.151	0.225	0.253	G:A
rs1837606	0.430	0.398	0.410	T:C
rs1871428	0.477	0.413	0.461	G:A
rs1871534	0.075	0.000	0.000	G:C
rs1876482	0.131	0.146	0.146	G:A
rs1879488	0.104	0.102	0.107	C:A
rs192655	0.175	0.253	0.267	A:G
rs1950993	0.467	0.383	0.348	G:T
rs2001907	0.051	0.092	0.045	C:T
rs200354	0.210	0.209	0.320	G:T
rs2024566	0.285	0.413	0.410	A:G
rs2030763	0.215	0.286	0.298	G:A
rs2033111	0.112	0.141	0.163	A:G
rs2042762	0.023	0.039	0.034	T:C
rs2070586	0.201	0.160	0.236	G:A
rs2073821	0.121	0.199	0.169	C:T
rs2125345	0.411	0.427	0.388	T:C
rs214678	0.070	0.068	0.056	T:C
rs2166624	0.364	0.340	0.354	G:A
rs2196051	0.290	0.347	0.381	G:A
rs2238151	0.311	0.471	0.343	C:T
rs2306040	0.096	0.082	0.101	T:C
rs2330442	0.411	0.427	0.348	A:G
rs2357442	0.262	0.238	0.331	A:C
rs2416791	0.159	0.121	0.174	G:A
rs2504853	0.467	0.409	0.379	T:C
rs2532060	0.439	0.413	0.427	C:T
rs2593595	0.355	0.233	0.303	A:G
rs260690	0.170	0.093	0.101	A:C
rs2627037	0.193	0.209	0.185	G:A
rs2702414	0.117	0.083	0.180	G:A
rs2814778	0.215	0.000	0.000	T:C
rs2835370	0.131	0.165	0.118	T:C
rs2899826	0.234	0.243	0.292	A:G
rs2946788	0.387	0.282	0.287	T:G
rs2966849	0.140	0.180	0.208	G:A
rs2986742	0.290	0.165	0.208	T:C
rs310644	0.178	0.140	0.296	T:C
rs3118378	0.234	0.257	0.348	A:G
rs316598	0.421	0.403	0.489	T:C
rs316873	0.131	0.180	0.225	C:T

Table S2 (continued)

	Minor allele frequency			
**Target ID**	**Baloch (n = 107)**	**Pashtun (n = 103)**	**Punjabi (n = 89)**	**Alleles (major : minor)**
rs32314	0.325	0.377	0.393	T:C
rs37369	0.178	0.206	0.236	C:T
rs3737576	0.028	0.034	0.034	T:C
rs3745099	0.280	0.320	0.236	A:G
rs3784230	0.217	0.272	0.281	A:G
rs3793451	0.047	0.083	0.079	C:T
rs3793791	0.089	0.112	0.157	T:C
rs3811801	0.000	0.000	0.000	G:G
rs3814134	0.093	0.034	0.101	A:G
rs3823159	0.042	0.070	0.090	A:G
rs3827760	0.005	0.015	0.006	A:G
rs385194	0.495	0.461	0.399	A:G
rs3907047	0.098	0.112	0.163	T:C
rs3916235	0.100	0.104	0.087	C:T
rs3943253	0.127	0.117	0.073	A:G
rs4411548	0.065	0.248	0.169	C:T
rs4458655	0.198	0.097	0.185	T:C
rs4463276	0.383	0.417	0.449	G:A
rs4471745	0.042	0.059	0.073	G:A
rs459920	0.288	0.422	0.287	T:C
rs4666200	0.266	0.248	0.264	A:G
rs4670767	0.106	0.191	0.258	G:T
rs4717865	0.215	0.189	0.270	G:A
rs4746136	0.236	0.218	0.236	G:A
rs4781011	0.327	0.267	0.360	G:T
rs4798812	0.383	0.291	0.386	G:A
rs4821004	0.416	0.456	0.371	C:T
rs4833103	0.019	0.042	0.039	C:A
rs4880436	0.005	0.015	0.011	C:T
rs4891825	0.154	0.146	0.124	A:G
rs4908343	0.346	0.296	0.382	A:G
rs4918664	0.168	0.214	0.360	A:G
rs4918842	0.182	0.097	0.112	T:C
rs4951629	0.037	0.039	0.034	T:C
rs4955316	0.140	0.121	0.157	T:G
rs4984913	0.360	0.495	0.494	A:G
rs5768007	0.285	0.257	0.348	C:T
rs6104567	0.192	0.330	0.437	T:G
rs6422347	0.224	0.126	0.219	T:C
rs6451722	0.297	0.321	0.202	G:A
rs6464211	0.220	0.243	0.264	C:T
rs647325	0.164	0.189	0.174	A:G

Table S2 (continued)

	Minor allele frequency			
**Target ID**	**Baloch (n = 107)**	**Pashtun (n = 103)**	**Punjabi (n = 89)**	**Alleles (major : minor)**
rs6541030	0.121	0.117	0.056	G:A
rs6548616	0.443	0.255	0.253	T:C
rs6556352	0.389	0.341	0.356	C:T
rs671	0.000	0.000	0.000	G:G
rs6754311	0.290	0.252	0.275	C:T
rs6990312	0.346	0.277	0.331	G:T
rs705308	0.374	0.257	0.258	A:C
rs7226659	0.056	0.092	0.073	G:T
rs7238445	0.329	0.282	0.315	G:A
rs7251928	0.206	0.180	0.219	A:C
rs731257	0.098	0.165	0.180	G:A
rs7326934	0.112	0.034	0.017	G:C
rs734873	0.114	0.206	0.180	G:A
rs735480	0.178	0.150	0.219	T:C
rs7421394	0.430	0.311	0.326	A:G
rs7554936	0.364	0.257	0.191	T:C
rs7657799	0.084	0.024	0.045	T:G
rs7722456	0.243	0.117	0.129	T:C
rs772262	0.229	0.126	0.163	G:A
rs7745461	0.292	0.214	0.264	G:A
rs7803075	0.425	0.427	0.489	A:G
rs7844723	0.332	0.382	0.354	C:T
rs798443	0.299	0.233	0.258	A:G
rs7997709	0.139	0.138	0.153	T:C
rs8021730	0.285	0.189	0.292	G:T
rs8035124	0.393	0.377	0.399	A:C
rs8113143	0.453	0.490	0.393	A:C
rs818386	0.075	0.103	0.129	C:T
rs870347	0.103	0.131	0.135	A:C
rs874299	0.500	0.461	0.494	C:T
rs881728	0.201	0.160	0.135	C:A
rs917115	0.294	0.335	0.410	T:C
rs9291090	0.079	0.131	0.107	A:C
rs9319336	0.121	0.160	0.163	T:C
rs946918	0.164	0.107	0.219	G:T
rs948028	0.304	0.155	0.185	A:C
rs9522149	0.374	0.408	0.292	T:C
rs9530435	0.201	0.136	0.124	C:T
rs9809104	0.285	0.170	0.174	T:C
rs9845457	0.463	0.408	0.399	A:G

[1]Variants marked in red were not considered in the estimation of the proportions of ancestry.

Table S3: Pairwise F_{ST}-values among the three Pakistani subpopulations Baloch, Pashtun, and Punjabi based on the allele frequencies of 165 AIMs typed with the Precision ID Ancestry panel.

Populations	Baloch	Pashtun	Punjabi
Baloch	-	*	*
Pashtun	0.0107	-	*
Punjabi	0.0117	0.0048	-

* The corresponding *p*-values were all below $p < 10^{-5}$ after Bonferroni correction.

Table S4: Allele frequencies of 163 variants typed with the Human Pigmentation (HuPi) AmpliSeq™ Custom panel in 299 Pakistani individuals from three subpopulations (Baloch, Pashtun, and Punjabi).

**Target ID[1]**	**Minor allele frequency**	**Alleles (major : minor)**
**rs12203592**	0.008	C:T
**rs4959270**	0.301	C:A
**rs401681**	0.242	C:T
**rs10424065**	0.005	C:T
**rs6510760**	0.189	G:A
**rs1042522**	0.500	C:G
**rs80293268**	0.000	G:C
**rs10809819**	0.058	A:T
rs10960748	0.277	A:C
rs75001364	0.017	A:G
**rs1408799**	0.329	T:C
**rs62538946**	0.135	A:G
rs112446852	0.018	A:G
rs79589462	0.131	A:T
rs10960751	0.273	T:C
rs10960752	0.273	G:A
rs74874037	0.017	T:C
**rs16929345**	0.070	G:C
**rs62538950**	0.103	A:T
rs13296454	0.273	G:A
**rs202148591**	0.017	AGGAG:AG
rs77448128	0.017	G:C
rs75611179	0.017	G:T
**rs144403042**	0.408	AC:-
**rs62538954**	0.089	G:C
rs62538956	0.090	T:C
rs10491745	0.287	C:T
**rs10756391**	0.289	C:T
rs77494858	0.017	C:A
**rs79586719**	0.017	G:A
rs62538957	0.085	G:A
rs78774349	0.017	C:T
rs80287758	0.015	G:A
rs111589749	0.017	T:C
rs76969096	0.018	C:G
rs112201749	0.017	C:T

Table S4 (continued)

**Target ID**[1]	**Minor allele frequency**	**Alleles (major : minor)**
rs77445059	0.014	C:A
rs117687305	0.018	T:C
rs74889423	0.017	G:A
rs11791497	0.017	A:C
rs11787674	0.017	G:A
rs11791954	0.017	A:T
**rs112923802**	0.000	TCTCTCTCTCTCTCTCTCTCTCTC:TCTCTCTCTCTCTCTCTCTCTCTC
rs76988967	0.017	C:A
rs62638049	0.017	A:T
rs61758391	0.017	G:A
**rs11787999**	0.017	C:T
rs34509359	0.017	C:A
**rs141808617**	0.017	AA:A
rs74606098	0.017	C:T
rs77446525	0.018	A:T
**rs139301549**	0.032	TTTTTT:TTTTT
rs35866166	0.017	T:C
rs112342609	0.018	G:T
rs77990455	0.017	T:A
**rs149076115**	0.017	AAGAGA:-
rs79662637	0.017	G:A
**rs59308154**	0.000	TTTTTTTTT:TTTTTTTTTT
rs17280279	0.018	G:A
**rs2733832**	0.252	C:T
rs2209278	0.018	C:T
rs683	0.283	C:A
**rs113819841**	0.017	AAAAA:AAAA
**rs10756819**	0.386	A:G
**rs12350739**	0.204	G:A
**rs6475555**	0.313	A:G
**rs199355**	0.350	G:A
**rs1545397**	0.089	A:T
**rs1800414**	0.007	T:C
**rs74653330**	0.002	C:T
**rs121918166**	0.000	C:T
**rs1800407**	0.050	C:T
**rs12441727**	0.195	G:A
**rs1448484**	0.072	A:G
**rs1470608**	0.386	G:T
**rs4778241**	0.338	A:C

Table S4 (continued)

Target ID[1]	Minor allele frequency	Alleles (major : minor)
**rs7495174**	0.286	A:G
rs1129038	0.100	C:T
**rs12913832**	0.100	A:G
**rs2238289**	0.480	G:A
**rs6497292**	0.306	A:G
**rs1667394**	0.294	C:T
**rs111458470**	0.379	-:CAG
**rs4424881**	0.258	C:T
**rs16891982**	0.191	C:G
**rs28777**	0.326	C:A
**rs26722**	0.237	C:T
**rs13289**	0.335	C:G
**rs6059655**	0.018	G:A
**rs4911414**	0.144	G:T
**rs1015362**	0.176	C:T
**rs6119471**	0.018	C:G
**rs2424984**	0.197	T:C
rs6058017	0.199	A:G
**rs2378249**	0.276	A:G
**rs4911442**	0.055	A:G
**rs2284063**	0.450	A:G
**rs4821767**	0.376	C:A
**rs117612447**	0.005	C:T
**rs1544410**	0.467	C:T
**rs2228570**	0.259	G:A
**rs1426654**	0.118	A:G
rs2470102	0.115	A:G
**rs11614913**	0.356	C:T
**rs73132911**	0.034	T:C
**rs3733542**	0.113	G:C
**rs12668421**	0.299	A:T
**rs11238349**	0.294	G:A
**rs3829241**	0.149	G:A
**rs36118030**	0.000	G:A
**rs2276288**	0.406	A:T
**rs1279403**	0.428	C:T
**rs10831496**	0.421	G:A
**rs12821256**	0.013	T:C
**rs1042602**	0.149	C:A
**rs1393350**	0.108	G:A

Table S4 (continued)

**Target ID[1]**	**Minor allele frequency**	**Alleles (major : minor)**
rs1126809	0.100	G:A
**rs3114908**	0.286	C:T
**rs258322**	0.070	G:A
**rs3212355**	0.000	C:T
**rs312262906**	0.000	A:AA
**rs1805005**	0.020	G:T
**rs1805006**	0.000	C:A
**rs2228479**	0.033	G:A
**rs11547464**	0.000	G:A
**rs1805007**	0.007	C:T
**rs201326893**	0.000	C:A
**rs1110400**	0.000	T:C
**rs1805008**	0.012	C:T
**rs885479**	0.042	G:A
**rs1805009**	0.000	G:C
**rs8051733**	0.237	A:G
**rs8059973**	0.176	G:A
**rs12896399**	0.308	G:T
**rs2402130**	0.186	A:G
**rs34755843**	0.003	CGACTCTCGA:CGA
**rs4904887**	0.462	G:C
**rs12590749**	0.448	A:C
**rs4904891**	0.216	G:C
**rs4904897**	0.154	C:T
**rs17128288**	0.196	A:G
**rs17128291**	0.054	A:G
**rs7152962**	0.360	G:A
**rs59977926**	0.291	T:C
rs35617057	0.468	G:T
rs12894869	0.466	C:G
rs12895667	0.477	A:G
rs7142428	0.463	C:T
rs4410008	0.473	C:T
rs7143110	0.473	C:G
**rs7144261**	0.473	A:T
**rs7144273**	0.492	T:C
**rs12894551**	0.306	C:T
**rs78863223**	0.219	A:G
**rs10131374**	0.202	G:A
**rs17128324**	0.196	C:T

Table S4 (continued)

**Target ID**[1]	**Minor allele frequency**	**Alleles (major : minor)**
**rs12880508**	0.253	T:C
**rs4904927**	0.149	G:A
**rs11160071**	0.190	C:T
**rs2031526**	0.303	G:A
**rs2369633**	0.043	C:T
**rs12623857**	0.054	G:A
**rs6742078**	0.371	G:T

[1]Independent variants are marked in bold. Variants not considered in the correlation analyses are marked in red.

Table S5: Estimated ancestry proportions associated with skin pigmentation on the upper arm, lower arm, or forehead in 299 Pakistani individuals from three subpopulations (Baloch, Pashtun, and Punjabi).

	Upper arm		Lower arm		Forehead	
	Adjusted R^2	Sign.[1]	Adjusted R^2	Sign.[1]	Adjusted R^2	Sign.[1]
Europe	0.04	*	0.09	**	0.04	*
South-Central Asia	< 0.01	-	0.02	-	< 0.01	-
Sub-Saharan Africa	0.06	*	0.03	-	0.07	**
East Asia	< 0.01	-	< 0.01	-	< 0.01	-
Full ancestry profile[2]	0.10	**	0.13	***	0.12	***
Europe + Sub-Saharan Africa	0.10	**	0.13	***	0.12	***

[1]Statistical significance (Sign.) after Bonferroni correction: *** $p < 10^{-6}$, ** $p < 10^{-3}$, * $p < 0.05$.

[2]Proportions of European, South-Central Asian, Sub-Saharan African, and East Asian ancestry.
